# Torsion of parietal-peritoneal fat mimicking acute appendicitis: a case report

**DOI:** 10.1186/1752-1947-3-6980

**Published:** 2009-04-27

**Authors:** Kamal Sanjiva Hapuarachchi, Edward Douglas Courtney, Szabolcs Gergely, Tjun Yip Tang

**Affiliations:** 1 Hinchingbrooke Hospital, Hinchingbrooke Park, Huntingdon, PE29 6NT, UK; 2 University Department of Radiology, Cambridge University Hospitals NHS Foundation Trust, Hills Road, Cambridge, CB2 2QQ, UK

## Abstract

**Introduction:**

Infarctions of the greater omentum and appendices epiploicae are uncommon, but well documented causes of acute abdominal pain. We present a rare case of torted fat on the parietal peritoneum over the anterior abdominal wall, mimicking clinical signs of acute appendicitis, which was diagnosed at laparoscopy. We are aware of only two other similar reported cases, both of which were diagnosed at the time of laparotomy.

**Case presentation:**

A 41-year-old Caucasian woman presented with clinical signs of acute appendicitis. On diagnostic laparoscopy, a non-inflamed appendix was found. Further exploration revealed a necrotic torted appendage of fat overlying the parietal peritoneum of the right iliac fossa of the anterior abdominal wall.

**Conclusion:**

Torted fatty appendages can be a diagnostic dilemma often mimicking more common causes of an acute abdomen. Laparoscopy is an excellent tool making the correct diagnosis in such cases.

## Introduction

Appendices epiploicae are small pouches of fat-filled peritoneum, which protrude from the serosal surface of the colon, and are usually arranged in two separate longitudinal rows extending from the caecum to the recto-sigmoid junction. They are typically between 1 and 2 cm wide and 2 to 5 cm long, and approximately 50 to 100 are present on average. They are usually supplied via their stalk by one or two arterioles from the vasa recta of the colon and drained by a single tortuous venule [1]. Appendices epiploicae can undergo ischaemia and localized inflammation due to either spontaneous torsion leading to compromise of their blood supply or venous thrombosis of the draining appendageal vein.

Infarctions of appendices epiploicae and the greater omentum are uncommon, but well documented causes of acute abdominal pain. Torsion of intraperitoneal fat on the parietal peritoneum is an even rarer phenomenon, with only two previously reported cases, both of which were found at laparotomy [2,3]. One case occurred in a 20-year-old Russian woman with a 12-hour history of right-sided abdominal pain and peritonism, and exploratory laparotomy revealed a 3 cm × 2 cm necrotic piece of fat on the parietal peritoneum 10 cm to the right of the umbilicus [2]. The other case occurred in a 25-year-old African-American man with a three-day history of symptoms and signs suggestive of appendicitis, but the patient was subsequently found to have a normal appendix at open appendicectomy. Further exploration revealed an infarcted appendage of fat suspended on the parietal peritoneum of the anterior abdominal wall at the level of the lateral border of the caecum [3].

## Case presentation

**Figure 1:**
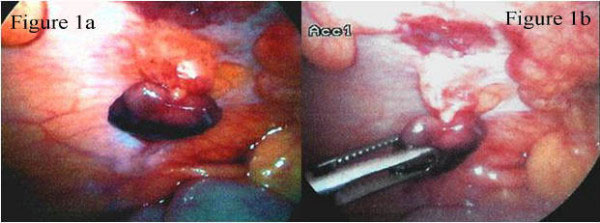
**Peritoneal fat appendage twisted on its pedicle on the anterior abdominal wall seen during diagnostic laparoscopy (a) and its subsequent removal (b)**.

A 41-year-old Caucasian woman presented with a one-day history of progressively worsening right iliac fossa pain. The pain was constant and made worse on movement. She had no change in her bowel habit and complained of nausea but no vomiting. Urinalysis was normal and a urinary pregnancy test was negative. On clinical examination, she had a mild pyrexia (37.5 °C), and abdominal examination revealed marked tenderness over the right iliac fossa with signs of localized peritonism. Blood tests were normal apart from a slightly elevated white cell count of 12.2 × 10^9^/L (neutrophil count 8.1 × 10^9^/L). A diagnosis of acute appendicitis was suspected and a diagnostic laparoscopy was performed. At laparoscopy, a non-inflamed appendix was seen and there was no free intraperitoneal fluid. No gynaecological pathology was seen. On the anterior abdominal wall above the right iliac fossa was a 3 cm × 2 cm piece of fat adherent to the peritoneum via a pedicle, around which it had torted (Figure 1). The fat was necrotic and was excised laparoscopically. An appendicectomy was not undertaken. The patient's pain resolved immediately post-operatively and she was discharged home the following day. Histology confirmed the macroscopic findings of fat necrosis.

## Discussion

The aetiology of such fatty pedicles overlying the peritoneum is somewhat of a mystery. The anatomist Richard Snell was unable to offer any embryological explanation for such a finding on personnel communication with Perry and Hawksley [3]. Extraperitoneal fat herniating through the peritoneum is a possibility, but no such defect was found in any of the cases. Harrigan [4] credited Virchow as the first person to describe appendices epiploicae presenting as loose peritoneal bodies. Virchow showed that obesity or infection resulted in increased fat deposition in the appendices epiploicae, which then undergo saponification and calcification, resulting in an increase in their weight. Progressive obliteration of the vessels within the pedicle occurs until necrosis ensues and the necrotic, calcified appendix epiploica becomes free within the peritoneal cavity [4]. In rare instances, it may reattach to an adjacent surface in which case it is termed a parasitized appendix epiploica. However, this is an unlikely aetiology for fat on the parietal peritoneum in our patient due to the lack of calcification on histology.

## Conclusion

Torsion of intraperitoneal fat is a rare but well recognised cause of acute abdominal pain, often mimicking more common causes of an acute abdomen such as appendicitis, diverticulitis and cholecystitis. It has been estimated that abdominal fat necrosis including omental torsion accounts for 1.1% of patients presenting with abdominal pain [5]. Whilst the diagnosis can be made pre-operatively by computed tomography scanning, the majority of cases are still diagnosed intra-operatively. Laparoscopy is an excellent diagnostic tool in the management of acute abdominal pain, allowing inspection of the entire abdominal cavity, and ensuring that less common causes of abdominal pain are correctly diagnosed and treated.

## Consent

Written informed consent was obtained from the patient for publication of this case report and accompanying images. A copy of the written consent is available for review by the Editor-in-Chief of this journal.

## Competing interests

The authors declare that they have no competing interests.

## Authors' contributions

KSH was responsible for the first draft of the manuscript. KSH, EDC and TYT were responsible for reviewing subsequent drafts and approving the final draft. All authors were involved with the General Surgery Department medical care of the patient. EDC was the primary surgeon and KSH was the assisting surgeon during the laparoscopy establishing the diagnosis. SG was the consultant surgeon responsible for the care of the patient.
